# Clinical Trial Publication Trends Within Neurology

**DOI:** 10.1515/tnsci-2019-0037

**Published:** 2019-08-20

**Authors:** Phan Q. Duy, Anirudh Sreekrishnan, Wyatt David, Manish D. Paranjpe, Ishan Paranjpe, Amar Sheth, Batur Gültekin, Kevin N. Sheth

**Affiliations:** 1 Medical Scientist Training Program, Yale University School of Medicine, New Haven, CT, USA; 2 Harvard Neurology Program, Brigham and Women’s Hospital & Massachusetts General Hospital, Boston, MA, USA; 3 Harvard-MIT Program in Health Sciences and Technology, Harvard Medical School, Boston, MA, USA; 4 Icahn School of Medicine at Mt. Sinai, New York, NY, USA; 5 Department of Neurology, Yale University School of Medicine, New Haven, CT, USA

**Keywords:** clinical trials, neurology, clinical research, neuroscience, clinicaltrials.gov

## Abstract

Timely dissemination of results from clinical studies is crucial for the advancement of knowledge and clinical decision making. A large body of research has shown that up to half of clinical trials do not publish their findings. In this study, we sought to determine whether clinical trial publication rates within neurology have increased over time. Focusing on neurology clinical trials completed between 2008 to 2014, we found that while the overall percentage of published trials has not changed (remaining at approximately 50%), time to publication has significantly decreased. Our findings suggest that clinical trials within neurology are being published in a more timely manner.

## Introduction

Clinical trials are an integral part of translating scientific findings and improve patient outcomes. Thus, dissemination of clinical trials results is crucial for informing public health policy and clinical decision making. However, dissemination rates of clinical trials results are disappointing: 25-50% of clinical trial results are not published [1, 2]. Within the field of neurology, we previously found that approximately only half of clinical trials published their findings in peer-reviewed journals [3]. In recognition of this major public health dilemma, governmental policies have been implemented to improve dissemination of clinical research results, including the 2007 US FDA Amendment Act (FDAAA) mandating clinical trial registration and results reporting on ClinicalTrials.gov a publicly accessible clinical registry, for all trials of FDA-regulated products [4, 5]. In this study, we sought to expand upon our previous findings to characterize trends in publication of clinical trials within the field of neurology. Specifically, we sought to determine: 1) Trends in the percentage of completed trials that published their findings from 2008 to 2014, and 2) Trends in the overall time to publication following trial completion.

## Methods

Data analysis was conducted using a database that we generated [3]. Briefly, we performed a search on July 19, 2016, through ClinicalTrials. gov for interventional trials conducted within the United States between 2007 and 2014 using the search term “nervous system disease.” Publication status was verified using the associated ClinicalTrials.gov webpage and the SCOPUS (Elsevier) database. We first grouped neurology clinical trials into the years when they were completed (2008 to 2014). For each year, we calculated the percentage of completed clinical trials that published their findings and the time to publication (defined as months between publication date and the primary completion date). To analyze trends in percent of completed trials that published their findings and time to publication, we used the Chi-Square test for trend and the Ordinary one-way ANOVA test followed by Tukey’s multiple comparisons test, respectively. All statistical tests were conducted using GraphPad Prism.

## Results

**Figure 1:**
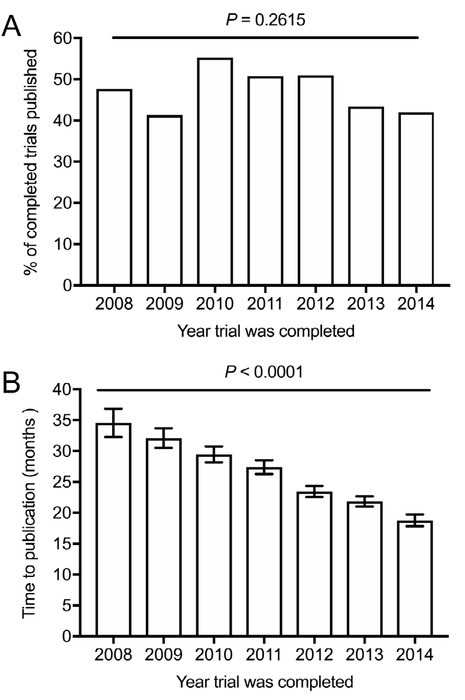
Clinical trial publication trends within neurology. A) Trends in percentage of published neurology clinical trials from 2008 to 2014. Chi-square test for trend was used to determine the p value. B) Trends in time to publication (in months between publication date and primary completion date) of neurology clinical trials from 2008 to 2014. One-way ANOVA test was used to determine the p value. Mean ± SEM are shown for each year.

Table 1 shows the number of completed and published trials per year from 2008 to 2014. Overall, we did not find any trends in percentage of published clinical trials from 2008 to 2014, as the publication rates per year remained at approximately 50% (p = 0.2615) (Figure 1A) For published trials, we found that the overall time to publication decreased from 2008 -2014 (p < 0.0001) (Figure 1B) For example, time to publication for published trials completed in 2008 was 34.59 ± 2.30 months, whereas time to publication for published trials completed in 2014 was only 18.79 ± 0.95 months (p < 0.0001) (Figure 1B)

**Table 1:** Number of completed and published trials per year from 2008 to 2014.

Year	Number of completed trials within year	Number of published trials within year
2008	172	82
2009	242	100
2010	284	157
2011	358	182
2012	406	207
2013	394	171
2014	193	81

## Discussion

Clinical trials are crucial for the translation of scientific discoveries into diverse areas of clinical medicine. Thus, timely dissemination of clinical trial results is important for the scientific progress by informing future research and medical practice. We found that while the overall percentage of clinical trials that published their findings has not changed, the time to publication has significantly decreased from 2008 to 2014. These findings suggest that despite no overall improvements in publication rates, clinical trials in neurology are being published in a more timely manner following study completion.

Although the scientific and medical communities have now recognized low publication of clinical trials to be a major problem [2, 6], the reasons underlying the failure to report clinical trial results are not understood. In a study that extensively reviewed controlled clinicals in fragile X syndrome [7], the most translated neurodevelopmental disorder [8], some large well-powered clinical trial studies remain unpublished, while others took some time to be published as they showed “failed” primary outcomes. The difficulty in publishing trials with inconclusive and negative results may thus explain why approximately half of neurology clinical trials are not published [3]. Indeed, clinical trials with positive findings were more likely to be recommended for publication compared to those with negative findings [9]. However, regardless of outcomes, timely reporting of all research findings is an important duty to the public and to patients that researchers need to uphold so that clinical science can progress [6]. Future investigations should seek to determine the underlying mechanisms that explain low rates of publication in clinical trials and thus help to define better research practice policies to ensure timely dissemination of clinical trial results.

## References

[1] Ross J.S. (2013). Time to publication among completed clinical trials. JAMA Intern Med.

[2] Chen R. (2016). Publication and reporting of clinical trial results: cross sectional analysis across academic medical centers. BMJ.

[3] Sreekrishnan A. (2018). Publication and Dissemination of Results in Clinical Trials of Neurology. JAMA Neurol.

[4] Phillips A.T. (2017). Association of the FDA Amendment Act with trial registration, publication, and outcome reporting. Trials.

[5] (2007). The Food and Drug Administration Amendments Act (FDAAA). Public Law: 110-85.

[6] Wallach J.D., Krumholz H.M. (2019). Not Reporting Results of a Clinical Trial Is Academic Misconduct. Ann Intern Med.

[7] Budimirovic D.B. (2017). Updated report on tools to measure outcomes of clinical trials in fragile X syndrome. J Neurodev Disord.

[8] Duy P.Q., Budimirovic D.B. (2017). Fragile X Syndrome: Lessons Learned from the Most Translated Neurodevelopmental Disorder in Clinical Trials. Transl Neurosci.

[9] Emerson G.B. (2010). Testing for the presence of positive-outcome bias in peer review: a randomized controlled trial. Arch Intern Med.

